# Previous COVID-19 Infection and Antibody Levels After Vaccination

**DOI:** 10.3389/fpubh.2021.778243

**Published:** 2021-12-01

**Authors:** Hamad Ali, Barrak Alahmad, Abdullah A. Al-Shammari, Abdulmohsen Alterki, Maha Hammad, Preethi Cherian, Irina Alkhairi, Sardar Sindhu, Thangavel Alphonse Thanaraj, Anwar Mohammad, Ghazi Alghanim, Sriraman Deverajan, Rasheed Ahmad, Sherief El-Shazly, Ali A. Dashti, Mohammad Shehab, Salman Al-Sabah, Abdullah Alkandari, Jehad Abubaker, Mohamed Abu-Farha, Fahd Al-Mulla

**Affiliations:** ^1^Department of Medical Laboratory Sciences, Faculty of Allied Health Sciences, Health Sciences Center (HSC), Kuwait University, Jabriya, Kuwait; ^2^Department of Genetics and Bioinformatics, Dasman Diabetes Institute (DDI), Dasman, Kuwait; ^3^Department of Environmental Health, Harvard T. H. Chan School of Public Health, Harvard University, Boston, MA, United States; ^4^Department of Mathematics, Faculty of Sciences, Kuwait University, Khaldiya, Kuwait; ^5^Department of Otolaryngology, Head and Neck Surgery, Zain and Al-Sabah Hospitals, Ministry of Health, Kuwait City, Kuwait; ^6^Medical Division, Dasman Diabetes Institute (DDI), Dasman, Kuwait; ^7^Department of Immunology and Microbiology, Dasman Diabetes Institute (DDI), Dasman, Kuwait; ^8^Department of Biochemistry and Molecular Biology, Dasman Diabetes Institute (DDI), Dasman, Kuwait; ^9^National Dasman Diabetes Biobank, Dasman Diabetes Institute (DDI), Dasman, Kuwait; ^10^Gastroenterology Unit, Department of Internal Medicine, Mubarak Al-Kabeer Hospital, Ministry of Health, Kuwait City, Kuwait; ^11^COVID-19 Research Group, Jaber Al-Ahmad Al-Sabah Hospital, Kuwait City, Kuwait

**Keywords:** COVID-19, SARS-CoV-2, BNT162b2, antibodies, humoral response, ChAdOx1, COVID-19 infection

## Abstract

**Background:** The emergence of new COVID-19 variants of concern coupled with a global inequity in vaccine access and distribution has prompted many public health authorities to circumvent the vaccine shortages by altering vaccination protocols and prioritizing persons at high risk. Individuals with previous COVID-19 infection may not have been prioritized due to existing humoral immunity.

**Objective:** We aimed to study the association between previous COVID-19 infection and antibody levels after COVID-19 vaccination.

**Methods:** A serological analysis to measure SARS-CoV-2 immunoglobulin (Ig)G, IgA, and neutralizing antibodies was performed on individuals who received one or two doses of either BNT162b2 or ChAdOx1 vaccines in Kuwait. A Student *t*-test was performed and followed by generalized linear regression models adjusted for individual characteristics and comorbidities were fitted to compare the average levels of IgG and neutralizing antibodies between vaccinated individuals with and without previous COVID-19 infection.

**Results:** A total of 1,025 individuals were recruited. The mean levels of IgG, IgA, and neutralizing antibodies were higher in vaccinated subjects with previous COVID-19 infections than in those without previous infection. Regression analysis showed a steeper slope of decline for IgG and neutralizing antibodies in vaccinated individuals without previous COVID-19 infection compared to those with previous COVID-19 infection.

**Conclusion:** Previous COVID-19 infection appeared to elicit robust and sustained levels of SARS-CoV-2 antibodies in vaccinated individuals. Given the inconsistent supply of COVID-19 vaccines in many countries due to inequities in global distribution, our results suggest that even greater efforts should be made to vaccinate more people, especially individuals without previous COVID-19 infection.

## Introduction

The coronavirus disease 2019 (COVID-19) pandemic continues to affect global health at an unprecedented scale with >200 million cases and >4 million deaths worldwide to date, with ongoing strains on the global economy (1). Non-pharmaceutical interventions have been implemented worldwide since the outbreak started in attempts to slow the pandemic surges, and efforts have been focused on the development of effective COVID-19 vaccines to curtail the pandemic, especially among vulnerable subpopulations (2–6).

The World Health Organization and the US Food and Drug Administration (FDA) had declared the release of COVID-19 vaccines in September 2020 (7). Between December 2020 and February 2021, adenoviral vector vaccines, such as ChAdOx1 (AstraZeneca-Oxford) and mRNA vaccines, such as BNT162b2 (Pfizer–BioNTech) and mRNA-1273 (Moderna) were put in use. The three vaccines are ChAdOx1, which consists of a non-replicative simian adenovirus vector with the full-length code of the spike protein of severe acute respiratory syndrome coronavirus 2 (SARS-CoV-2) and the other two, which utilize a novel mRNA vaccine platform, have shown acceptable safety and efficacy profiles in clinical trials (8–10). Remarkably, these vaccines, which are administered in two intramuscular shots, have been shown to offer protection by stimulating production of anti-S-protein receptor binding domain (S-RBD) immunoglobulin G (IgG), IgM, and IgA isotypes, with neutralization activity capable of inhibiting binding of RBD to the angiotensin-converting enzyme 2 (ACE2) cognate receptor (11, 12). Measurement of these antibodies in sera, particularly the neutralizing antibody levels, can indicate the level of protection induced by either COVID-19 vaccination or previous infection (13).

There is a strong debate concerning the nature, stability, and durability of antibody responses over time in COVID-19 patients, with several studies reporting stable antibody persistent immunity (14) and others showing rapidly waning antibody immunity, or late appearance with low antibody levels, and/or complete lack of long-lasting antibodies (15, 16). Further studies are needed to demonstrate the safety and efficacy of booster vaccinations, especially in immunocompromised and/or immunosuppressed individuals to determine the best dosing schedule and mix-and-match schedules of vaccinations. US public health officials have urged the FDA to decide on booster vaccines because the benefits of COVID-19 vaccination far outweigh the potential risks (17). Several countries have announced plans for booster-shot programs (18). Third doses of vaccines developed by Moderna, Pfizer–BioNTech, Oxford–AstraZeneca, and Sinovac have prompted a spike in levels of infection-blocking “neutralizing” antibodies when administered several months after the second dose (18).

On the other hand, previously infected individuals have been shown to have some humoral protection against COVID-19 (19), although still prone to reinfection (20, 21). Calls for global vaccine equity continue to fail as many developing countries are incapable of obtaining enough vaccines to protect a substantial proportion of the population. Toward this end, many countries have attempted to ration their vaccine supplies by prioritizing individuals who were not previously infected. In Kuwait, individuals who had previous COVID-19 infections were temporarily given a single dose of vaccine, whereas those who had not had the infection were prioritized to receive two doses. We then compared the levels of humoral antibody response between the two groups.

This study specifically compared the antibody-mediated immune response in terms of SARS-CoV-2 S1-specific IgG, IgA, IgM, and anti-S RBD neutralizing antibody levels between vaccinated people who had previous COVID-19 infections with the response in vaccinated people who had not had COVID-19 infections in Kuwait.

## Materials and Methods

### Participant's Recruitment

The Ethical Review Committee of the Dasman Diabetes Institute and Ethical Committee of the Kuwait Ministry of Health reviewed and approved the study per the updated guidelines of the Declaration of Helsinki and of the US Federal Policy for the Protection of Human Subjects (approval references; RA HM-2021-008 and 3799, respectively). The study recruited people who received either one or two doses of the BNT162b2 (Pfizer–BioNTech) mRNA vaccine and ChAdOx1 (AstraZeneca) vaccine. The two groups were further divided into subgroups on the basis of with or without previous COVID-19 infection. The diagnosis of COVID-19 was established on the basis of a positive SARS-CoV2 polymerase chain reaction result for a nasopharyngeal swab. Participants receiving immunosuppressants and/or chemotherapy were excluded from the study. A RedCap survey was used to collect data from each participant and included age, sex, chronic health conditions, height, weight, and self-reported history of COVID-19 infection. Samples were collected at the Dasman Diabetes Institute after signed informed consent was obtained from all participants.

### Blood Sample Collection

Blood samples were obtained from the participants and collected in EDTA tubes. Samples were centrifuged at 400 x g for 10 min at normal room temperature to separate the plasma, which was then aliquoted and stored at −80°C.

### Quantification of Plasma Levels of SARS-CoV-2-Specific IgG, IgM, and IgA

An enzyme-linked immunosorbent assay (ELISA) kit (SERION ELISA agile SARS-CoV-2 IgG and IgA SERION Diagnostics, Würzburg, Germany) were used to determine plasma levels of SARS-CoV-2-specific IgG and IgA antibodies following the manufacturers' protocols. The positive and negative cutoffs were determined per the manufacturers' recommendations. The IgG levels were reported as binding antibody units (BAU)/mL. Values <21 BAU/mL were considered to be negative, values from 21.0 to 31.5 BAU/mL were considered to be borderline, and levels >1.5 BAU/mL were considered to be positive. The IgM levels were reported as Arbitrary Units (AU)/mL. Values <90 AU/mL were considered to be negative, values from 90 to 110 AU/mL were considered to be borderline, and values >110 AU/mL were considered to be positive. The IgA levels were reported as AU/mL. Values <10 AU/mL were considered to be negative, values from 10 to 14 AU/mL were considered to be borderline, and values >14 AU/mL were considered to be positive.

### Quantification of Plasma Levels of SARS-Cov-2-Specific Neutralizing Antibodies

A SARS-CoV-2-specific surrogate Virus Neutralization Test (sVNT) was used to study the levels of plasma neutralizing antibodies against SARS-CoV-2 S-RBD (SARS-Cov-2 sVNT kit, GenScript, USA, Inc.) according to the manufacturer's protocol. Evaluation of the data was performed by following the manufacturer's recommendations for positive and negative cutoffs. The results were obtained by calculating inhibition rates for samples per the following equation: Inhibition = (1 – sample O.D./O.D. negative control) × 100%. Neutralizing antibody levels >20% were considered to be positive.

### Statistical Analysis

The descriptive data were summarized as the mean, median, standard deviation, and interquartile range where applicable. For the regression analysis, we restricted the data to individuals who received both doses of BNT162b2. Mean levels of IgG and neutralizing antibodies were initially compared between vaccinated individuals with and without previous COVID-19 infection using Student *t*-test. Due to potential confounding by multiple factors, inferences from such analyses should be interpreted with caution. We attempted to address this potential confounding by building generalized additive linear models in which plasma IgG and neutralizing antibody levels were fit as the dependent variables, adjusting for type-2 diabetes status (yes/no), hypertension (yes/no), body mass index (BMI) (linear), age (smoothed spline), sex, comorbidity score (sum of the scores with equal weighting for heart disease, stroke, chronic obstructive pulmonary disease, asthma, obstructive sleep apnea, chronic kidney disease, bleeding disorders, and other chronic diseases), previous COVID-19 infection (yes/no), and duration since receiving the last vaccine dose (smoothed spline) as *a priori* independent variables. Penalized splines were fit for two continuous variables, age, and duration since last dose to explore and control for non-linearity in a restricted maximum likelihood estimation (22, 23). The smoothing penalized splines are non-parametric terms used to optimize the goodness-of-fit by cross-validation through a penalty term for over- or under-fitting. We determined and interpreted the adjusted effect estimates with 95% confidence intervals (CIs) as changes in the mean IgG levels by comparing (1) individuals with previous infection to those without and (2) the estimated decline for every 100 days since receiving the second dose. We further performed interaction analyses by assessing the effect of a previous infection on the slope of IgG decline over time. We included an interaction term between previous COVID-19 infection status and the duration (in days) since receiving the second to assess the effect measure modification and reported the Wald-test *p*-value for statistical significance. All analyses were performed by using R software version 3.3.1 (R Foundation for Statistical Computing), and penalized splines were implemented in generalized additive models by using the *mgcv* package. A *p* < 0.05 was considered to be indicative of statistical significance.

## Results

### Cohort Characteristics

The study included 1,025 participants who were descriptively divided into four groups according to the number of doses received and type of vaccine. Forty-one subjects received one dose of BNT162b2, 490 subjects received two doses of BNT162b2, 299 subjects received one dose of ChAdOx1, and 195 subjects received two doses of ChAdOx1. Each group was subdivided into two subgroups according to previous infection status. The distribution of the participants and their ages in each group are presented in Tables 1, 2.

**Table 1:** Cohort vaccination status stratified by previous COVID-19 infection.

	**No previous infection** **(*N* = 822)**	**Previous infection** **(*N* = 203)**	**Overall** **(*N* = 1,025)**
**Status**			
1 dose BNT162b2	14 (1.6%)	27 (11.2%)	41 (3.7%)
1 dose ChAdOx1	237 (27.8%)	62 (25.7%)	299 (27.3%)
2 doses BNT162b2	412 (48.2%)	78 (32.4%)	490 (44.7%)
2 doses ChAdOx1	159 (18.6%)	36 (14.9%)	195 (17.8%)

**Table 2:** Cohort characteristics and serological results.

	**1 dose BNT162b2**		**1 dose ChAdOx1**		**2 doses BNT162b2**		**2 doses ChAdOx1**		**Overall**	
	**No previous** **infection** **(*N* = 14)**	**Previous** **infection (*N* = 27)**	**No previous** **infection** **(*N* = 237)**	**Previous** **infection** **(*N* = 62)**	**No previous** **infection** **(*N* = 412)**	**Previous infection** **(*N* = 78)**	**No previous** **infection** **(*N* = 159)**	**Previous infection** **(*N* = 36)**	**No previous** **infection** **(*N* = 822)**	**Previous infection** **(*N* = 203)**
**Age (Years)**										
Mean (SD)	47.6 (12.6)	42.3 (11.6)	48.3 (11.0)	46.3 (9.24)	48.1 (14.2)	46.0 (13.8)	43.1 (11.4)	40.4 (10.3)	47.2 (12.9)	44.6 (11.8)
Median [Min, Max]	44.3 [21.5, 66.0]	42.4 [22.5, 73.2]	51.1 [21.0, 74.0]	47.5 [24.3, 63.6]	48.3 [21.8, 87.4]	42.7 [21.0, 74.2]	42.5 [21.0, 89.1]	38.0 [21.5, 65.0]	47.6 [21.0, 89.1]	42.9 [21.0, 74.2]
**Duration (Days)**										
Mean (SD)	31.8 (19.3)	56.7 (31.2)	91.6 (25.6)	99.4 (27.1)	91.7 (45.1)	82.1 (45.4)	30.0 (12.4)	28.9 (11.9)	78.7 (43.3)	74.6 (42.2)
Median [Min, Max]	22.0 [7.00, 76.0]	52.0 [9.00, 124]	99.0 [19.0, 150]	105 [21.0, 159]	90.0 [6.00, 197]	76.0 [12.0, 195]	26.0 [1.00, 61.0]	27.0 [5.00, 48.0]	78.0 [1.00, 197]	72.0 [5.00, 195]
**IgG (BAU/ml)**										
Mean (SD)	157 (63.5)	195 (39.9)	80.0 (70.1)	155 (61.2)	137 (55.1)	188 (42.7)	116 (50.5)	146 (42.2)	117 (64.1)	172 (52.1)
Median [Min, Max]	172 [5.10, 226]	204 [67.6, 263]	51.6 [1.42, 261]	177 [5.85, 230]	141 [11.0, 260]	198 [35.5, 266]	116 [0, 245]	155 [5.94, 209]	122 [0, 261]	185 [5.85, 266]
Missing	0 (0%)	0 (0%)	0 (0%)	1 (1.6%)	0 (0%)	0 (0%)	0 (0%)	0 (0%)	0 (0%)	1 (0.5%)
*P*-value	0.009		<0.001		<0.001		<0.001		<0.001	
**Neutralizing antibodies (%)**										
Mean (SD)	76.9 (25.8)	90.8 (7.14)	49.6 (35.9)	83.1 (24.1)	82.2 (16.1)	91.0 (10.7)	82.8 (18.7)	90.6 (12.8)	72.7 (28.4)	88.4 (16.5)
Median [Min, Max]	86.8 [3.70, 94.3]	93.5 [65.5, 95.3]	38.7 [0, 95.1]	93.7 [10.3, 95.3]	88.7 [0, 95.1]	94.1 [28.2, 95.8]	90.4 [3.92, 95.1]	93.4 [22.4, 95.1]	87.2 [0, 95.1]	93.7 [10.3, 95.8]
Missing	0 (0%)	1 (3.7%)	2 (0.8%)	1 (1.6%)	17 (4.1%)	8 (10.3%)	3 (1.9%)	3 (8.3%)	22 (2.7%)	13 (6.4%)
*P*-value	0.017		<0.001		<0.001		0.761		<0.001	
**IgM (AU/ml)**										
Mean (SD)	141 (201)	126 (123)	39.7 (70.0)	58.6 (78.9)	50.7 (76.0)	79.6 (88.4)	32.5 (67.5)	42.3 (50.7)	45.5 (77.7)	72.8 (88.8)
Median [Min, Max]	71.9 [5.50, 800]	81.7 [6.70, 407]	13.3 [0, 494]	30.0 [0.800, 474]	23.6 [0, 600]	50.4 [6.50, 600]	15.7 [1.43, 551]	24.2 [5.07, 250]	18.8 [0, 800]	40.4 [0.800, 600]
*P*-value	0.78		0.055		0.006		0.01		<0.001	
**IgA (AU/ml)**										
Mean (SD)	30.5 (29.2)	66.1 (40.9)	11.5 (22.5)	41.6 (42.9)	22.8 (34.5)	69.2 (48.3)	11.6 (20.9)	31.9 (26.1)	17.5 (29.5)	53.8 (44.8)
Median [Min, Max]	19.6 [3.34, 102]	55.7 [14.3, 155]	2.78 [0.0400, 200]	27.0 [1.19, 200]	9.42 [0, 200]	58.3 [0, 200]	4.17 [0.151, 130]	25.8 [0.422, 93.9]	6.29 [0, 200]	38.1 [0, 200]
Missing	2 (14.3%)	3 (11.1%)	34 (14.3%)	12 (19.4%)	38 (9.2%)	8 (10.3%)	2 (1.3%)	1 (2.8%)	76 (9.2%)	24 (11.8%)
*P*-value	0.015		<0.001		<0.001		<0.001		<0.001	

### Levels of Antibodies According to Previous Infection Status

**Figure 1:**
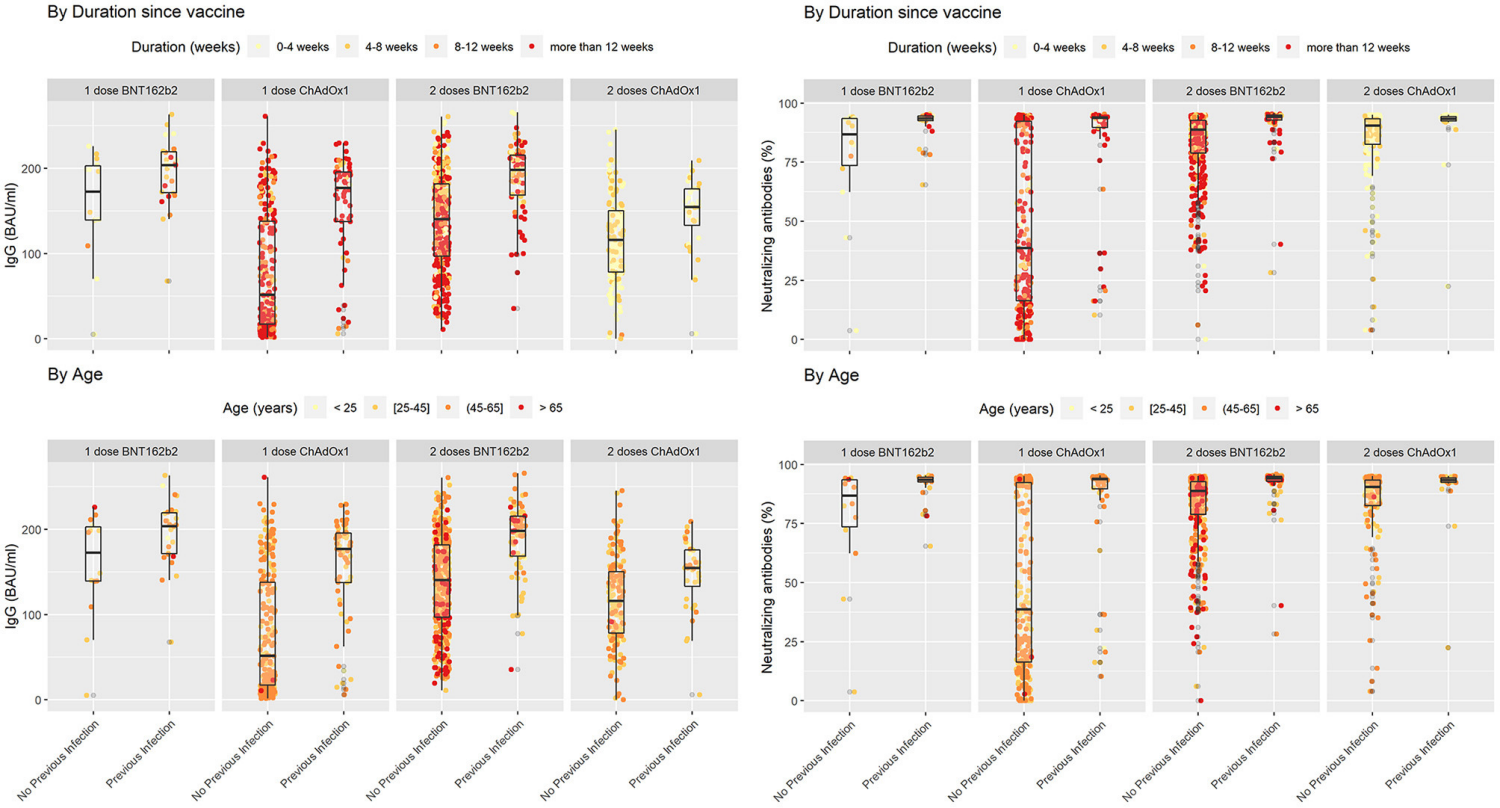
SARS-COV2 IgG and neutralizing antibodies in vaccinated individuals stratified by previous infection status while duration since vaccination and age variations shown based on color coding. In each subgroup individuals with previous COVID-19 infection showed higher levels of IgG and Neutralizing antibodies in comparison with their counterparts who did not encounter COVID-19. Lower levels of IgG seem to be associated with a cluster of older individuals who received two doses of BNT162b2 without previous infection.

**Figure 2:**
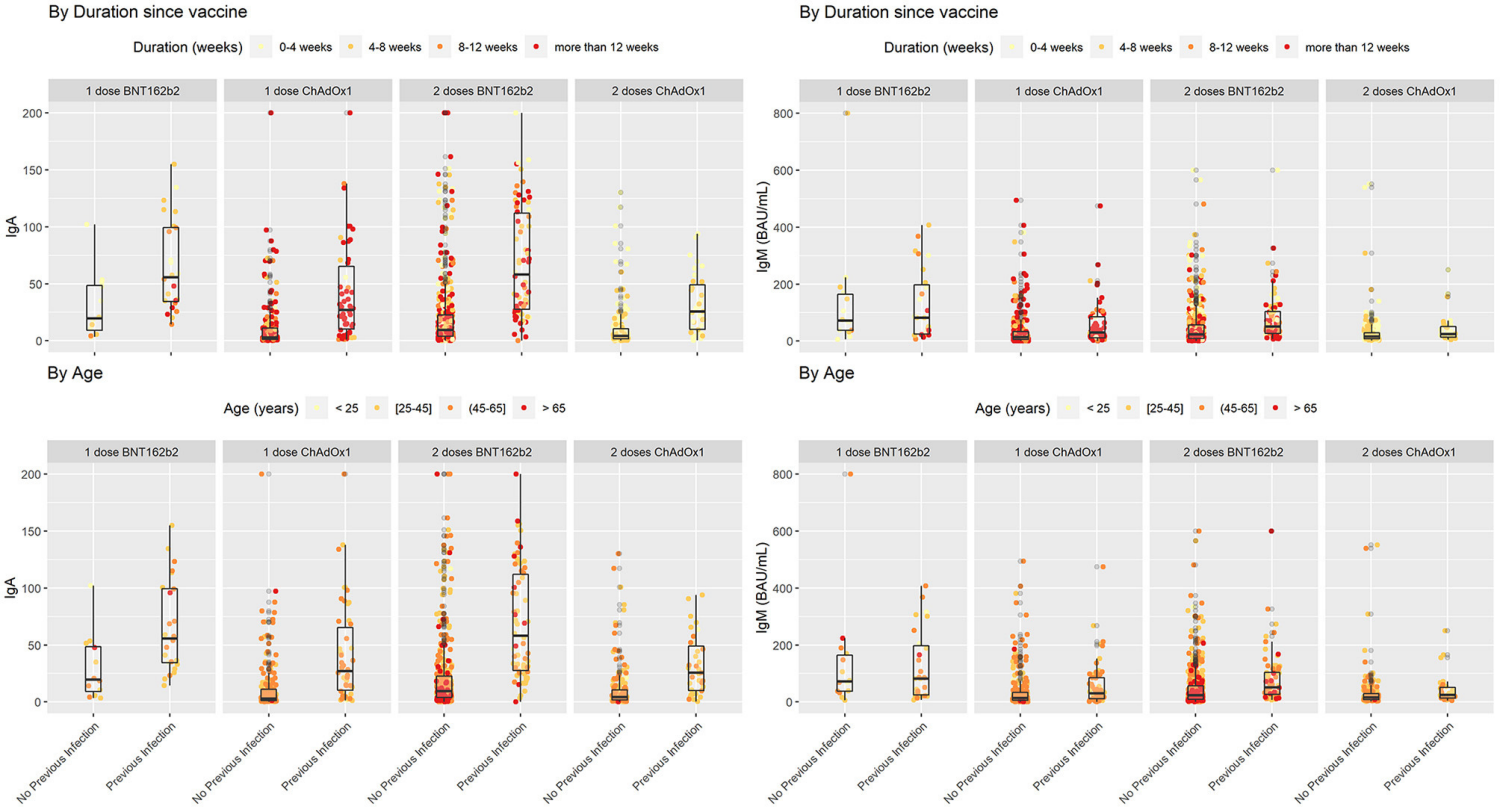
SARS-COV2 IgA and IgM antibodies in vaccinated individuals stratified by previous infection status while duration since vaccination and age variations shown based on color coding. In each subgroup individuals with previous COVID-19 infection showed higher levels of IgA in comparison with their counterparts who did not encounter COVID-19. Lower levels of IgA seem to be associated with a cluster of older individuals who received two doses of BNT162b2 without previous infection.

We descriptively compared mean levels of SARS-CoV-2 IgG, IgM, IgA, and neutralizing antibodies in the groups based on COVID-19 previous infection status. In each group, the mean levels of SARS-CoV-2 IgG, IgA, and neutralizing antibodies were higher in the subgroups of vaccinated subjects with previous COVID-19 infection than in those without previous COVID-19 infection (Table 2) and (Figures 1, 2). The mean duration between the serology test and last dose of vaccine taken was 74.6 ± 42.3 days for subjects with previous infection and 78.7 ± 43.3 days for those without previous infection (Table 2).

In people who took one dose of BNT162b2 the mean level of SARS-CoV-2 IgG antibodies was 157 ± 63.5 BAU/mL in vaccinated individuals who were not infected with SARS-CoV2 compared with people with previous COVID-19 infection 195 ± 39.9 BAU/mL (*P* = 0.009). In the same group the mean percentage of neutralizing antibodies was 76.9 ± 25.8% in people without previous SARS-CoV2 infection compared to 90.8 ± 7.14% in those with previous SARS-CoV2 infection (*P* = 0.017). The mean level of IgM was 141 ± 201 AU/mL in people without previous infection compared to 126 ± 123 AU/mL in those with previous COVID-19 infection (*P* = 0.78). Finally, level of IgA antibodies in vaccinated people with one dose of BNT162b2 was 30.5 ± 29.2 AU/mL in people without SARS-CoV2 infection vs. 66.1 ± 40.9 in people who were previously infected with SARS-CoV2 (*P* = 0.015) (Table 2).

In the second group which included people who took one dose of ChAdOx1, the mean level of IgG antibodies was 80.0 ± 70.1 BAU/mL in vaccinated individuals who were not infected with SARS-CoV2 compared with people with previous COVID-19 infection 155 ± 61.2 BAU/mL (*P* < 0.001). In the same group the mean percentage of neutralizing antibodies was 49.6 ± 35.9% in people without previous SARS-CoV2 infection compared to 83.1 ± 24.1% in those with previous SARS-COV2 infection (*P* < 0.001). The mean level of IgM was 39.7 ± 70.0 AU/mL in people without previous infection compared to 58.6 ± 78.9 AU/mL in those with previous COVID-19 infection (*P* = 0.055). Finally, level of IgA antibodies was 11.5 ± 22.5 AU/mL in people without SARS-CoV2 infection vs. 41.6 ± 42.9 in people who were previously infected with SARS-CoV2 (*P* < 0.001) (Table 2).

Participants that took two doses of the BNT162b2 or ChAdOx1 vaccine and were previously infected with SARS-CoV2 had a significantly higher levels of IgG, neutralizing, IgM, and IGA antibodies compared to participants who were not previously infected with SARS-CoV2. In people that took two doses of the BNT162b2 vaccine, IgG antibody level in people without previous infection was 137 ± 55.1 vs. 188 ± 42.7 BAU/mL in people with previous SARS-CoV2 (*P* < 0.001). IgG antibody level for people that took two doses of ChAdOx1 was 116 ± 50.5 BAU/mL in people without previous infection vs. 146 ± 42.2 BAU/mL in people with previous SARS-CoV2 infection (*P* < 0.001). on the other hand, the mean percentage of neutralizing antibodies was 82.2 ± 16.1% in people who took two doses of BNT162b2 without previous SARS-CoV2 infection compared to 91.0 ± 10.7% in those with previous SARS-CoV2 infection (*P* < 0.001). For ChAdOx1 vaccine, this level was 82.8 ± 12.8% in people who took two doses without previous SARS-CoV2 infection compared to 90.6 ± 12.8% in those with previous SARS-CoV2 infection (*P* = 0.761).

Individuals who took two doses of BNT162b2 had a mean level of IgM of 50.7 ± 76.0 AU/mL if they were not previously infected with SARS-CoV2 compared to 79.6 ± 88.4 AU/mL in those with previous COVID-19 infection (*P* = 0.006). IgA antibodies in vaccinated people with two doses of BNT162b2 was 22.8 ± 34.5 AU/mL in people without SARS-CoV2 infection vs. 69.2 ± 48.3 in people who were previously infected with SARS-CoV2 (*P* < 0.001) (Table 2). Finally, IgM level in people who took two doses of ChAdOx1 was 32.5 ± 67.5 AU/mL if they were not previously infected with SARS-CoV2 compared to 42.3 ± 50.7 AU/mL in those with previous COVID-19 infection (*P* = 0.01). IgA antibody level in this group was 11.6 ± 20.9 AU/mL in people without SARS-CoV2 infection vs. 31.9 ± 26.1 in people who were previously infected with SARS-CoV2 (*P* < 0.001) (Table 2).

Overall, the mean level of IgG was 172 ± 52.1 BAU/mL in vaccinated individuals who received one or two doses of either vaccine with previous COVID-19 infection and 117 ± 64.1 BAU/mL in those without previous COVID-19 infection (Table 2). The mean level of IgA was 53.8 ± 44.8 AU/mL in vaccinated individuals who received one or two doses of either vaccine with previous COVID-19 infection and 17.5 ± 29.5 AU/mL in those without previous COVID-19 infection (Table 2). The mean percentage of neutralizing antibodies was 88.4 ± 16.5% in vaccinated individuals who received one or two doses of either vaccine with previous COVID-19 infection and 72.7 ± 28.4% in those without previous COVID-19 infection (Table 2). More descriptive data comparing antibody levels across BNT162b2 or ChAdOx1 recipients are provided in Supplementary Table 1.

### Regression Analyses

**Figure 3:**
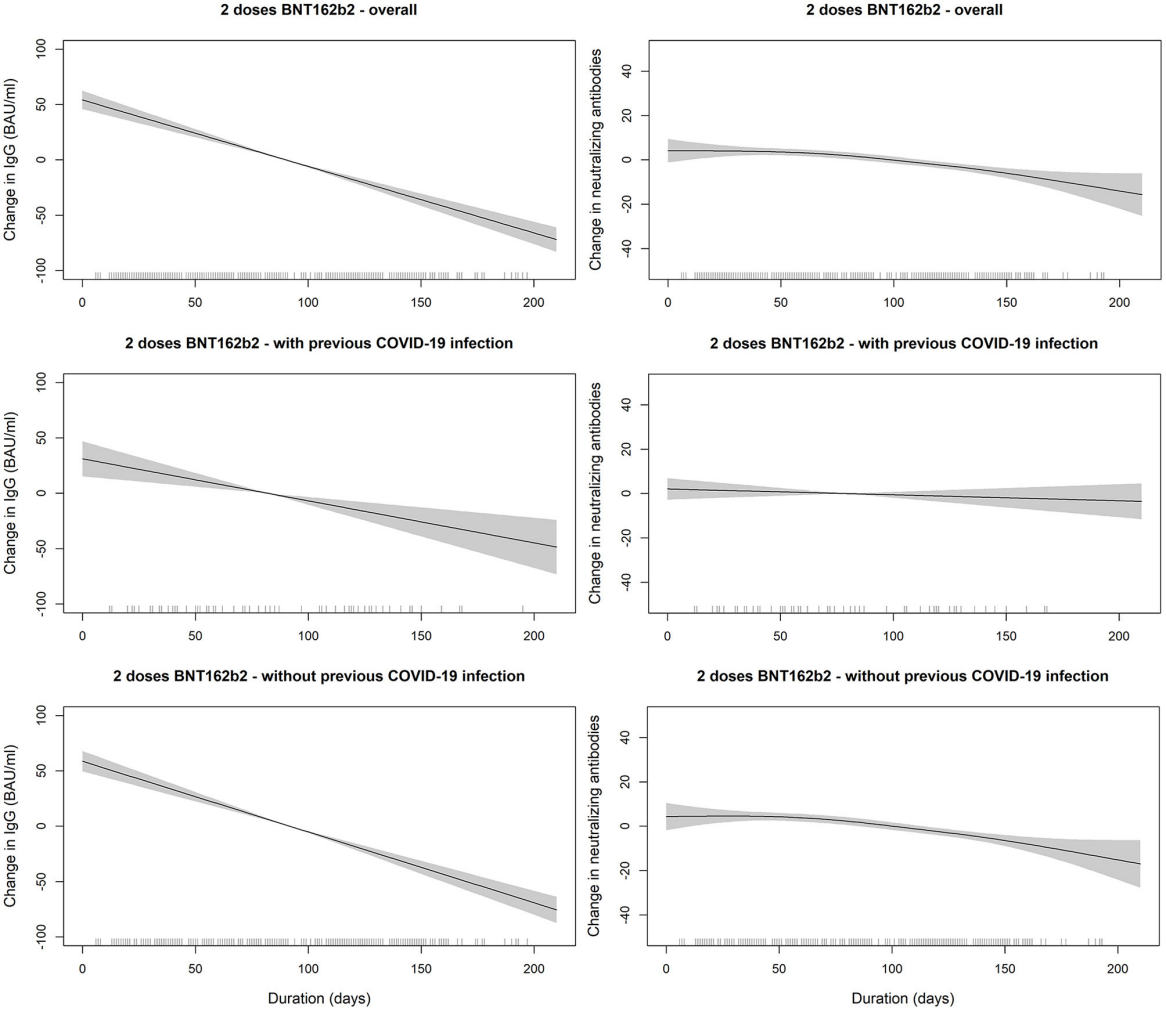
SARS-COV2 IgG and neutralizing antibodies decline over time since receiving the second dose of BNT162b2 (Pfizer–BioNTech) vaccine stratified by previous COVID-19 infection status. The smooth relationships were derived from generalized additive models with penalized splines for duration (in days) and adjusted for age, gender, BMI, and comorbidity scores. Solid lines represent the effect estimates of change over time while the shaded areas represent the 95% confidence intervals. The graphs show faster decline for both markers in vaccinated individuals without previous COVID-19 infection in comparison to those with previous infection.

Generally, IgG declined linearly as more days passed after receiving the vaccine dose. Average IgG levels declined by −60.05 BAU/mL (95% CI; −68.98, −51.11; *p* < 0.001) for every 100 days passed since the second vaccine (Table 3). IgG duration-adjusted smooth relationships with 95% CIs are presented in Figure 3. Among vaccinated individuals, those with previous COVID-19 infection had, on average, higher IgG levels by 46.65 BAU/mL (95% CI; 35.85, 57.45; *p* < 0.001).

**Table 3:** Change in IgG levels (in BAU/ml) in adjusted multiple linear models for those who received 2 doses of BNT162b2.

**Variable**	**Change**	**95% low**	**95% high**	***p*-value**
**Overall**				
Previously infected vs. No previous infection	46.65	35.85	57.45	<0.001
Decline for every 100 days	−60.05	−68.98	−51.11	<0.001
**Interaction**				
Decline for every 100 days and no previous infection	−63.40	−73.09	−53.71	*p*-value for interaction = 0.084
Decline for every 100 days and previously infected	−42.28	−64.31	−20.25	

When the linear decreases in subjects receiving two doses of BNT162b2 were compared between those with previous COVID-19 infection vs. those without, we found that the IgG slope of the group without infection was steeper (Table 3). In the interaction analyses, IgG levels were lower in the group with two doses of BNT162b2 without previous infection than in the group with previous infection (*p*-value for interaction = 0.084) (Table 3). However, the interaction terms were likely not powered sufficiently to give statistically significant *p*-values.

## Discussion

Unquestionably, the best strategy to fight the COVID-19 pandemic is a global vaccination campaign able to administer safe and effective vaccines. However, this strategy has been challenged by inequitable vaccine distribution and access across countries. The COVID-19 vaccination campaign started in Kuwait in 2021 with priority given to high-risk groups, including health care workers, the elderly, and immunocompromised patients with pre-existing conditions. With the COVID-19 vaccine supply-chain problems in matching the increasing demands, health authorities were obliged to develop vaccination strategies to cope with inconsistent supply of COVID-19 vaccines. One approach was to include a single-dose provision to individuals who were previously infected in Kuwait. We relied on observational data from self-recruitment to measure antibody levels in Kuwait. Due to the limited data and the differential vaccine type received, we were not able to compare a single dose in individuals with infection to those with no previous infection and two doses. However, we indirectly investigated the effect of a previous COVID-19 infection on the SARS-CoV-2 antibodies among all individuals who completed two doses of vaccination. Such findings have the potential to help in vaccination prioritization strategies to cope with any shortage of vaccine supply.

The main finding of this study was that there were significantly higher levels of antibodies in fully-vaccinated individuals with previous COVID-19 infection (natural immunity) than in fully-vaccinated individuals without prior infection (acquired immunity). Additionally, we found that those without previous infection showed a faster decline in antibodies over time, a result also reported by previous studies (24). This difference based on infection status was not surprising and could be attributed to several factors. First, we still do not have a definite answer on how long antibodies would last after a COVID-19 infection or vaccination. Many studies are still detecting antibodies after several months of infection regardless of the severity of the disease, with one study showing detectable levels of neutralizing antibodies 7 months post-infection (25, 26). Similarly, studies on vaccines are showing a strong antibody response. A clinical trial on the Moderna vaccine reported high levels of antibodies 6 months after administering the second dose (27). Another study on ChAdOx1 showed high levels of antibodies 3 months after a single dose (28). Considering that the antibody-making B cells multiply after each exposure, whether due to the infection or the vaccine, the high antibody levels in the previously infected groups most likely represent the sum of the antibodies produced after the infection and after vaccination. Second, although the vaccines work by eliciting an immune response similar to that after a viral infection, they present the viral protein in slightly different conformations (29). In addition, and specifically for BNT162b2, mRNA delivery using lipid nanoparticles could present the antigens to the immune system in a different way than that in an actual viral infection, which could result in differences in antigen kinetics and the antibodies produced (30, 31). Finally, natural immunity and acquired immunity can differ in the types of antibodies produced. COVID-19 vaccines expose the immune system only to a certain part of the virus. Therefore, the immune system might not be producing as many different types of antibodies after vaccination as it would after an actual COVID-19 infection. The same reasons can be used to explain the observation that the levels of antibodies after a previous infection with one dose of either vaccine were higher than that after two doses without a previous infection.

In line with our findings, some studies have reported similar observations in smaller cohorts. For example, a study in 51 health care workers from the UK showed that previously infected individuals expressed higher levels of SARS-CoV-2 anti-S antibody titers than individuals with no previous COVID-19 infection after a single dose of BNT162b2 (32). In another study that was also conducted in health care workers, the authors observed that participants with prior infection had antibody titers one order of magnitude higher than those without a previous infection, and this was not affected by ethnicity or sex (33). In another study on BNT162b2, the authors reported similar IgG antibody levels and ACE2 antibody-binding inhibition responses between individuals with prior COVID-19 infection after a single dose and individuals without prior infection after two doses of vaccine (34). An interesting study that also reported conclusions similar to those reported here was conducted in individuals with autoimmune rheumatic diseases who were taking immunosuppressants (35). The study showed that one dose of ChAdOx1 in individuals with a previous infection was also superior to two doses in this subset of immunocompromised patients. Altogether, data from the present study combined with findings from previous work suggest that COVID-19 infection results in a broader immune response (Supplementary Table 1).

Our data clearly show the higher levels of IgG and neutralizing antibodies in the individuals with a previous infection and one dose of either vaccine compared to the completely vaccinated group with two doses of vaccine and no previous infection. In light with the increased demand and shortage of vaccine supply, this observation would support implementing a strategy of a single vaccine dose for individuals who were previously infected by SARS-CoV-2. Nonetheless, a follow-up study is eminent to determine the ideal timing for the administration of the second dose for this group by performing regular serological analysis and monitoring the decline in the levels of antibodies over time. In fact, a possible approach would be to perform such serological tests in the future for all individuals regardless of their vaccination/infection history to determine the need for booster shots.

The decline in IgG levels over time was expected since this occurs for all other vaccinations. There is still the question of how long these antibodies remain reasonably effective. The serological analysis in this study was performed an average of <3 months after vaccination. This finding just confirms previous findings showing that antibodies last for several months after vaccination (36–38). Additionally, we observed that the linear decline in IgG levels over time was faster among individuals who were not previously infected than in those who were infected (24). Although this can be explained by the assumed additive amount of antibodies produced after the infection and after vaccination, this phenomenon should be investigated in a follow-up longitudinal epidemiological design in which the two groups can be fairly compared with little concern about time-invariant confounders, such as age, sex, and BMI.

Overall, the differences in the neutralizing antibodies levels in different groups were lower than the differences in the SARS-CoV-2 IgG antibody levels for the same subgroups. We speculate that the factors that can possibly influence overall changes in neutralizing and SARS-CoV-2 IgG titers may relate to individual differences in DCs antigen processing and presentation and induction of specific antibody expressing plasma cells as well as variable depletion kinetics of the effector/memory responses and induced antibody isotypes. In any case, further studies in larger, more cohesive, and homogeneously selected cohorts may help understand contributory roles of these factors in both immunity arms.

Although we are optimistic that the ongoing vaccination campaign should provide some control of disease spread, herd immunity might be difficult to achieve as current vaccines, although they will reduce the degree of morbidity and mortality, they will not prevent the spread of infection. Therefore, these vaccines will probably be seasonal, so proper protocols are needed. In case of limited vaccine supply, immunocompromised people and individuals who were not previously infected may need to be vaccinated more promptly as they may still be susceptible to infection after vaccination and have some risk of more serious disease. Overall, the guidance for urgently needed booster shots should require continuous monitoring of the antibody levels in vaccinated individuals to maintain a protective COVID-19 immune response.

The emergence of the Delta variant (B.1.617) and its fast spread around the globe poses a serious problem for achieving herd immunity as current vaccines appear limited in their ability to slow transmission of the Delta variant, the current dominant strain. This observation is supported by several reports highlighting breakthrough infections of Delta in fully-vaccinated individuals (39, 40). As the economic, political, and psychological burden of the pandemic has been significant, countries around the globe are opening up, and public health measures are being relaxed and disengaged. This less careful attitude might allow the Delta variant to circulate more effectively resulting in new global COVID-19 waves, paving the way for a new approach to, perhaps, achieve herd immunity through what can be described as hybrid immunity. On one hand, the current vaccines provide effective protection against severe COVID-19 infections and death, viral exposure, on the other hand, might provide the needed broader immunity to block further transmission and hence, in principle, provide the lacking factor to surge toward herd immunity.

This study had a number of limitations. First, we only descriptively compared the different sub-stratifications by vaccine type, number of doses, and previous infection. The data were not originally collected to investigate those differences limiting the ability of inferences given the study design. Second, we were not able to investigate the associations between decline in antibody levels over time among ChAdOx1 recipients. This was due disrupted vaccination protocols leading to the sparse intervals between the two doses and a short duration among those who very recently received ChAdOx1 second dose. Third, although the regression analyses were adjusted for a number of *a priori* confounders, we cannot rule out potential residual confounding by other variables, such as severity of chronic illness or duration since previous infection. Finally, the cross-sectional data are prone to confounding by time-invariant factors, such as individual characteristics. Longitudinal analyses of both humoral and cellular responses with longer follow-ups are warranted to carefully assess the duration of immunity after vaccination.

In conclusion, the findings from this study confirm that vaccination with prior COVID-19 infection results in a stronger antibody response than vaccination without prior infection. These findings can help in implementing strategies for vaccination policies under the current inequities in the global distribution of and access to vaccines, especially when deciding on which individuals should be prioritized for booster shots. Specifically, single dose vaccination will currently suffice in previously infected individuals and future longitudinal studies will determine if and when a second dose would be needed.

## Data Availability Statement

The raw data supporting the conclusions of this article will be made available by the authors upon request.

## Ethics Statement

The studies involving human participants were reviewed and approved by the Ethical Review Committee of the Dasman Diabetes Institute and Ethical Committee of the Kuwait Ministry of Health reviewed and approved the study per the updated guidelines of the Declaration of Helsinki and of the US Federal Policy for the Protection of Human Subjects (approval references; RA HM-2021-008 for ERC and 3799). The patients/participants provided their written informed consent to participate in this study.

## Author Contributions

HA and MA-F conceived and designed the analysis, researched data, and wrote the manuscript. BA and AA-S performed the analysis and generated the figures. AAlt, MHa, IA, and PC involved in sample collection and laboratory analysis. SD, GA, and AM involved in subjects' recruitment. SA-S, MS, SE-S, AD, and AAlk, involved in the clinical design of the study and participant referrals from other hospitals. SS, TT, and RA edited the manuscript, managed and coordinated responsibility for the research activity planning and execution. JA and FA-M contributed to the discussion and Funding acquisition. All authors contributed to the article and approved the submitted version.

## Funding

This Study was funded by Kuwait Foundation for the Advancement of Sciences (KFAS) grant (RA HM-2021-008).

## Conflict of Interest

The authors declare that the research was conducted in the absence of any commercial or financial relationships that could be construed as a potential conflict of interest.

## Publisher's Note

All claims expressed in this article are solely those of the authors and do not necessarily represent those of their affiliated organizations, or those of the publisher, the editors and the reviewers. Any product that may be evaluated in this article, or claim that may be made by its manufacturer, is not guaranteed or endorsed by the publisher.
