# Supplementary material for: Previous COVID-19 Infection and Antibody Levels After Vaccination
Source: Front Public Health. 2021 Dec 1;9:778243. doi: 10.3389/fpubh.2021.778243 (PMC8671167; doi:10.3389/fpubh.2021.778243)
Supplement: Supplementary file 1 [file Table_1.DOCX]

Comparing overall between 2 vaccine doses versus one vaccine dose and a history of previous COVID-19 infection, comparable neutralizing antibody levels were found as though significantly higher IgG levels were induced with a single vaccine dose in previously infected people. Comparing within the same vaccine type, there was no statistical difference between neutralizing antibodies levels between people that received 2 doses of ChAdox1 and those with previous infection receiving 1 dose of ChAdOx1. Nonetheless, significant difference in neutralizing antibodies was found comparing people with 2 doses of BNT162b2 with those with a history of past infection and received 1 dose of BNT162b2. In this group, significantly higher levels of both neutralizing and IgG antibodies were induced by a single vaccine (BNT162b2) dose versus 2 doses of BNT162b2 in people with no history of previous infection.

**Supplementary table. 1**

|  | **2 doses ChAdOx1** | **1 dose ChAdOx1 + Infection** | **P value** |
| --- | --- | --- | --- |
| **Neutralizing antibodies (%)** | 82.18 | 83.12 | 0.761 |
| **IgG (BAU/mL)** | 114.09 | 155.19 | <0.0001 |
| **IgM (AU/mL)** | 31.71 | 58.57 | 0.010 |
| **IgA (AU/mL)** | 11.16 | 41.55 | <0.0001 |
|  |  |  |  |
|  | **2 doses BNT162b2** | **1 dose BNT162b2 + Infection** |  |
| **Neutralizing antibodies (%)** | 81.84 | 90.94 | 0.004 |
| **IgG (BAU/mL)** | 136.41 | 196.48 | <0.0001 |
| **IgM (AU/mL)** | 50.79 | 122.92 | <0.0001 |
| **IgA (AU/mL)** | 23.44 | 64.04 | <0.0001 |
|  |  |  |  |
|  | **2 doses (Overall)** | **1 dose + Infection (Overall)** |  |
| **Neutralizing antibodies (%)** | 81.94 | 85.58 | 0.070 |
| **IgG (BAU/mL)** | 130.29 | 168.50 | <0.0001 |
| **IgM (AU/mL)** | 45.56 | 79.08 | <0.0001 |
| **IgA (AU/mL)** | 19.86 | 49.24 | <0.0001 |
|  |  |  |  |
|  | **2 doses BNT162b2** | **2 doses BNT162b2 + Infection** |  |
| **Neutralizing antibodies (%)** | 81.84 | 91 | <0.0001 |
| **IgG (BAU/mL)** | 136.41 | 188 | <0.0001 |
| **IgM (AU/mL)** | 50.79 | 79.6 | <0.0001 |
| **IgA (AU/mL)** | 23.44 | 69.2 | <0.0001 |
|  |  |  |  |
|  |  |  |  |
|  | **2 doses ChAdOx1** | **2 doses ChAdOx1 + Infection** |  |
| **Neutralizing antibodies (%)** | 82.80 | 90.6 | <0.0001 |
| **IgG (BAU/mL)** | 116.00 | 146 | <0.0001 |
| **IgM (AU/mL)** | 32.50 | 42.3 | <0.01 |
| **IgA (AU/mL)** | 11.60 | 31.9 | <0.0001 |
